# 

**Published:** 1903-07-15

**Authors:** 


					﻿THE
DENTAL REGISTER
EDITED BY
NELVILLE S. HOFF, D. D. S.,
ANN ARBOR, MICH.
Vol. LV1I.	JULY 15, 1903.	No. 7.
PRICE, ONE DOLL-A-R- A YEAR IN ADVANCE.
PUBLISHED MONTHLY BY
SAMUEL· A. CROCKER & CO.,
THE OHIO DENTAL AND SURGICAL DEPOT.
35 to	’W'. Stlx SSt., CineiniTfiti, O.
Entered at the Postoffice at Cincinnati, 0., as second-class mail matter.
				

